# Do endocrine adverse events predict longer progression-free survival among patients with non-small-cell lung cancer receiving nivolumab?

**DOI:** 10.1371/journal.pone.0257484

**Published:** 2021-09-29

**Authors:** Izabela Chmielewska, Marta Dudzińska, Michał Szczyrek, Joanna Świrska, Kamila Wojas-Krawczyk, Agnieszka Zwolak

**Affiliations:** 1 Department of Pneumology, Oncology and Allergology, Medical University of Lublin, Lublin, Poland; 2 Chair of Internal Medicine and Department of Internal Medicine in Nursing, Medical University of Lublin, Lublin, Poland; Baylor College of Medicine, UNITED STATES

## Abstract

The aim of the study was to assess the occurrence and nature of immune-related endocrine adverse events (irAEs) among patients with non-small-cell lung cancer (NSCLC) treated with nivolumab.

**Methods:** The study group included 35 patients (15 women, 20 men, 65.8 ± 7.1 years) with NSCLC in stage IIIB (n = 16, 45.7%) and IV (n = 19,54.3%) who were treated with nivolumab.

**Results:** Of the studied patients, 34.3% (n = 12) developed endocrine irAEs (irAE group): 22.9% (n = 8) hyperthyroidism and 8.6% (n = 3) hypothyroidism, and in one case, hypophysitis was observed. The median irAEs onset time was 2 months. In the group of patients with thyroid disorders, permanent hypothyroidism eventually developed in 58.3%. The severity of the analyzed irAEs ranged from mild to moderate (Grade 1–2); the case of hypophysitis was estimated as Grade 3. The comparison of progression-free survival time (PFS) between the two groups showed longer PFS in patients in the irAE group (p = 0.021). Patients with irAE were treated significantly longer with nivolumab and they received more doses of nivolumab, however in Cox analysis we did not find patients with irAE to experience progression later than patients without them.

**Conclusions:** Nivolumab therapy is associated with an increased risk of endocrine adverse effects, particularly thyroid dysfunction. Endocrine adverse effects can be successfully treated pharmacologically and usually do not require discontinuation of immunotherapy. The relationship between a better cancer prognosis in patients who developed endocrine irAE has not been found.

## Introduction

The immune checkpoint inhibitors (ICIs) are a new class of anti-cancer drugs that have already proved their efficacy, revolutionizing treatment in many types of cancer, e.g., melanoma, non-small-cell lung cancer (NSCLC), and renal cancer. Their mechanism of action is to restore the function of the immune system that was blocked by a cancer that affects T and B lymphocytes, natural killer (NK) cells, and macrophages [1].

Focusing on NSCLC, in Poland, NSCLC patients currently have access to the following ICIs and are only reimbursed for this indication: nivolumab and pembrolizumab (programmed cell death protein (PD-1) inhibitors) and atezolizumab (programmed cell death ligand 1 (PD-L1) inhibitor). For first-line treatment, pembrolizumab can be used in NSCLC patients with high expression of PD-L1 on tumor cells (≥50%). No combination of immunotherapy with chemotherapy is available for Polish patients. For second-line treatment, regardless of PD-L1 expression, two drugs can be used, nivolumab and atezolizumab, both for non-small-cell lung cancer [2].

Nivolumab, a monoclonal antibody that inhibits the PD-1 molecule, is primarily located on activated T lymphocytes as well as on NK cells, that is, on the immune system cells involved in the anti-cancer response [3, 4]. The interaction between PD-1 molecule and its ligand, PD-L1 or PD-L2 located on cancer cells, leads to inhibiting the activity of T lymphocytes and results in apoptosis of these cells. Therefore, blocking PD-1 with nivolumab leads to the restoration of T cells activation and to enhancing anti-cancer response [1, 5].

With the emergence of immunotherapy in oncological treatment, we started to observe adverse events different from those normally associated with classical chemotherapy. Those differences result from the effect of ICIs on the immune system and its stimulation, which leads to autoimmune reactions, especially in the endocrine system, gastrointestinal tract, skin, and respiratory system [5]. Tolerance of immunotherapy is usually better than classic cytostatic or other methods of conventional treatment in oncology [6], although complications Grade 3–5 in the Common Terminology Criteria for Adverse Events (CTCAE) classification [7] are reported [8].

Among patients treated with nivolumab, common adverse events such as rash, musculoskeletal pain, fatigue, pruritus, diarrhea, and nausea, are observed in approximately 20% of patients [9]. Adverse events potentially related to immunological mechanisms (immune-related adverse events (irAEs)) include skin rash, endocrine disorders (particularly thyroiditis), gastrointestinal tract disorders (colitis, hepatitis), pulmonary adverse events (pneumonitis), and nephritis, which occurred with a 10–20% frequency [10]. Rare adverse effects, noted in less than 1.0% of treated patients, include myocarditis, myositis and rhabdomyolysis, uveitis, pancreatitis, polymyalgia, immunological neuropathy, Guillain–Barré syndrome, pericarditis, sarcoid reactions, vasculitis, aplastic anemia, and myasthenic syndromes. irAEs may require immunosuppressive medication to suppress the excessive immune response [11].

Among the endocrinopathies, the most observed are thyroid dysfunctions: hyperthyroidism (0.8–15.3%) and hypothyroidism (2.6–10.1%) [5, 12–19]. Hypophysitis is rare during nivolumab treatment (0.1–0.6%) [5, 20]; it is particularly associated with anti-cytotoxic T-lymphocyte-associated protein 4 (anti-CTLA-4) therapy [21]. Other adverse effects include insufficiency of adrenal glands (0.8–1.9%) [5, 18, 19, 22] and type 1 diabetes (0.1–1.5%) [5, 15, 18, 23, 24], which are rare endocrine toxicities associated with nivolumab treatment, but can be life-threatening if not promptly recognized and treated. The mechanism of endocrine irAEs induced by targeting PD-1 including nivolumab remains unclear. Endocrine adverse effects are usually mild, reaching no higher than Grade 2 in the CTCAE classification [7], can be successfully treated, and do not usually require discontinuation of immunotherapy [25].

A recent analysis of patients with NSCLC treated with anty-PD1 inhibitors found a positive association between thyroid dysfunction and a possible improvement in survival in patients who developed this complication [25–27]. The aim of this study was to assess the occurrence and nature of endocrine adverse events during nivolumab immunotherapy in patients with NSCLC in a real life setting, as well as to answer the question as to whether the development of endocrine irAEs impacts the effectiveness of the treatment.

## Materials and methods

### Characteristic of the study group

The study group included 35 patients (15 women and 20 men, mean age 65.8, ± 7.1 years) with NSCLC in stage IIIB (n = 16, 45.7%) and IV (n = 19, 54.3%). Histopathology included squamous cell carcinoma (n = 19, 54.3%), adenocarcinoma (n = 13, 37.1%), and not-otherwise-specified (NOS) NSCLC (n = 3, 8.6%) treated with nivolumab. In this article, we present the data of all patients who were enrolled to an early open-access program of nivolumab treatment in second and further lines of therapy before it was widely accessible and reimbursed in Poland.

Eligibility criteria included histologically or cytologically confirmed NSCLC in stage IIIB or IV [28] after failure of first line platinum-based chemotherapy or, in the case of patients with *EGRF* gene mutation, after failure of treatment with tyrosine-kinase inhibitors. In one of the enrolled patients, the ALK rearrangement in the absence of PD1 expression was identified during immunotherapy. Yet, nivolumab treatment was continued because of good therapeutic effect; in the long term, complete remission was achieved [29]. All patients at the moment of treatment introduction were in well general condition (ECOG-0/1, according to Eastern Cooperative Oncology Group Performance Status) [30].

All patients started treatment with nivolumab from November 2016 to January 2017. Treatment was continued to disease progression, death, or unaccepted tolerance. Retrospectively, all data from the beginning of the treatment until January 2020 were collected, involving 36 months of follow-up. Nivolumab was administered intravenously every 2 weeks at a dose of 3 mg/kg, after modification of the characteristics of the medicinal product at a constant dose of 240 mg at two weeks intervals. The characteristics of the study group are shown in Table 1. This was a retrospective study of medical records, patients provided informed written consent to have data from their medical records used in research, in compliance with principles of Declaration of Helsinki according to agreement of local ethics committee.

**Table 1:** Patients characteristics.

Variable	Overall N = 35	Non-irAEs group n = 23	AE group n = 12	*p*-value
Sex^1^				0.537
Female	15 (42.9)	9 (39.1)	6 (50)	
Male	20 (57.1)	14 (60.9)	6 (50)	
Age^2^ (years)	65.83 (7.12)	65.87 (6.14)	65.75 (9.02)	0.565
Histology^1^				0.011
squamous	19 (54.3)	12 (52.2)	7 (58.3)	
adenocarcinoma	13 (37.1)	11 (47.8)	2 (16.7)	
not-otherwise-specified (NOS)	3 (8.6)	0 (0.0)	3 (25.0)	
Age at NSCLC diagnosis^2^	64.29 (6,72)	64.52 (5,82)	63.83 (8,46)	0.542
Time from NSCLC diagnosis to initiation of nivolumab^3^ (months)	12 (3–96)	12 (3–39)	13,5 (9–96)	0.572
Stage^1^				0.279
IIIb	16 (45.7)	9 (39.1)	7 (58.3)	
IV	19 (54.3)	14 (60.9)	5 (41.7)	
*EGFR* mutation^1^				0.125
no	31 (88.6)	19 (82.6)	12 (100.0)	
yes	4 (11.4)	4 (17.4)	0 (0.0)	
*ALK* rearrangment^1^				>0.999
no	34 (97.1)	22 (95.7)	12 (100.0)	
yes	1 (2.9)	1 (4.3)	0 (0.0)	
Bone metastasis^1^				0.380
no	28 (80.0)	17 (73.9)	11 (91.7)	
yes	7 (20.0)	6 (26.1)	1 (8.3)	
Metastases to parenchymal organs^1^				0.434
no	25 (71.4)	15 (65.2)	10 (83.3)	
yes	10 (28.6)	8 (34.8)	2 (16.7)	
Central nervous system metastases^1^				>0.999
no	31 (88.6)	20 (87.0)	11 (91.7)	
yes	4 (11.4)	3 (13.0)	1 (8.3)	
Current or previous smoker^1^				0.400
no	6 (17.1)	5 (21.7)	1 (8.3)	
yes	29 (82.9)	18 (78.3)	11 (91.7)	
Pack years^2^	24.14 (14.73)	22.61(15.58)	27.08 (13.05)	0.388
Previous radiotherapy^1^				0.070
no	14 (40.0)	12 (52.2)	2 (16.7)	
yes	21 (60.0)	11 (47.8)	10 (83.3)	
Line of treatment^1^				0.308
2	22 (62.9)	12 (52.2)	10 (83.3)	
3	10 (28.6)	8 (34.8)	2 (16.7)	
4	2 (5.7)	2 (8.7)	0 (0.0)	
5	1 (2.9)	1 (4.3)	0(0.0)	
Patients with history of other cancers^1^				
<5 years before immunotherapy				
no	33 (94.3)	22 (95.7)	11 (91.7)	> 0.999
yes	2 (5.7)	1 (4.3)	1 (8.3)	
5–10 years before immunotherapy				0.343
no	34 (97.1)	23 (100)	11 (91.7)	
yes	1 (2.9)	0 (0.0)	1 (8.3)	
>10 years before immunotherapy				> 0.999
no	33 (94.3)	22 (95.7)	11 (91.7)	
yes	2 (5.7)	1 (4.3)	1 (8.3)	
% PD-L1 expresion^1^				> 0.999
PD-L1 ≥ 50	2 (12.5)	1 (8.3)	1 (25.0)	
PD-L1 1–49	5 (31.3)	4 (33.3)	1 (25.0)	
Unknown	9 (56.3)	7 (58.3)	2 (50.0)	
Median total doses of nivolumab^3^^,^ ^4^	5(1–76)	4 (1–66)	29 (3–76)	0.009
PFS (months)^3^^,^ ^5^	2.5 (1.50–10.0)	2 (1.50–2.50)	9.0 (3.50–26.0)	0.0214
OS (months)^5^	36.0 (32.00–50.0)	33.5 (20.0–40.50)	54.5 (34.0–65.0)	0.0953
Reason for termination of treatment^1^				0.459
Not applicable (ongoing treatment)	3 (8.5)	0 (0.0)	3 (25.0)	
Disease progression	29 (82.9)	22 (95.7)	7 (58.3)	
Adverse events	2 (5.7)	1 (4.3)	1 (8.3)	
Other	1 (2.9)	0 (0.0)	1 (8.3)	

M, mean; Me, median; SD, standard deviation

^1^ n (%)

^2^ mean (SD)

^3^ median (range minimum–maximum)

^4^ Mann–Whitney U test

^5^Kaplan-Meier analysis

AEs, adverse events; irAEs, immune-related adverse events; PFS, progression-free survival.

### Assessments of laboratory parameters

Thyroid function was assessed at baseline (before starting nivolumab) and every 4 weeks during treatment. We assessed the occurrence of adverse events affecting hormonal functions with a possible immunological mechanism (immune-related adverse events (irAEs)) related to nivolumab treatment. Adverse events were evaluated according to CTCAE v5.0 [7]. Subclinical hypothyroidism was defined as elevated thyroid-stimulating hormone (TSH) with normal free thyroxine (FT4) and free triiodothyronine (FT3) levels, and overt hypothyroidism was defined by elevated TSH with low FT4 and/or FT3. Hyperthyroidism was defined as a TSH concentration <0.1 mIU/L together with an elevated or normal free thyroid hormone. Thyroiditis/thyrotoxicosis was defined by the presence of a reduced level of TSH (<0.1 mIU/L) with normal or elevated FT4 or FT3 that spontaneously resolved or converted into hypothyroidism. When thyroid disorders were reported, thyroid autoantibodies: thyrotropin receptor antibodies (TRAbs), anti-thyroid peroxidase antibodies (TPOAbs), thyroglobulin antibodies (TGAbs) were measured and thyroid imaging (thyroid ultrasonography) was performed. For symptoms suggesting other endocrine disorders, appropriate diagnostics were widened. All patients with endocrine irAE induced by nivolumab were evaluated and treated by an endocrinologist. Laboratory tests (serum TSH, FT4, Ft3, TRAbs, TPOAbs, TGAbs, cortisol, adrenocorticotropin (ACTH), prolactin, luteinizing hormone (LH), follicle stimulating hormone (FSH), and estradiol) were evaluated using a chemiluminescent method (CLIA) with a Siemens Advia Centaur XP apparatus (New York, United States).

The time to occurrence of adverse events, recovery status, the use of systemic steroid therapy in the treatment of selected irAEs, and the impact of the presence of endocrine irAEs on the efficacy of lung cancer treatment were assessed. Patients were divided into two groups. The first comprised patients with endocrine-immunological adverse events (irAE group) and the second included patients without these effects (non-irAE group). In each group, we evaluated progression-free survival (PFS) and overall survival (OS) using the Kaplan–Meier method and log-rank test. Progression-free survival was defined as the time from start of treatment to documented disease progression or death. The irAE group included patients who had any hormonal disorders during immunotherapy (both de novo and change in severity or character of pre-existing endocrine disorders).

In addition, to reduce immortal time bias, a Cox regression model with time-dependent variables was used to verify whether subjects developing subsequent endocrine complications experienced cancer progression later.

In 48.5% of patients, we retrospectively determined PD-L1 expression in archived material using the immunohistochemical (IHC) method with the SP263 antibody clone in the BenchMark GX stain (Rotkreuz, Switzerland). We attempted to assess the association of endocrine irAEs occurrence with PD-L1 expression.

### Statistical analysis

The obtained results were subjected to statistical analysis. The values of the analyzed parameters measured on the nominal scale were characterized by the number and percentage, and on the quantitative scale by the mean, median, and standard deviation. To identify any differences a Mann–Whitney test or χ^2^ test was conducted. If the expected frequency in cells was too small, the Fisher’s exact test (for 2 × 2 tables) or Monte Carlo simulation (for larger tables) was performed. A 5% inference error and the related significance level *p* < 0.05, indicating the existence of statistically significant differences or dependencies, were assumed. Statistical analysis was performed in SPSS software version 25 (Predictive Solutions, Kraków, Poland).

## Results

### Underlying endocrine disorders and on treatment adverse events

Prior to initiating nivolumab treatment, all patients were euthyroid. Among the observed subjects, 14 patients had a prior history of endocrine diseases, including 5 patients diagnosed with nodular goiter. Four of them had no evidence of thyroid gland dysfunction during nivolumab therapy. However, in one of these patients, 2 months after treatment initiation, subclinical hyperthyroidism developed. The most likely mechanism of this abnormal finding was destructive thyroiditis (thyrotoxicosis).

**Fig 1:**
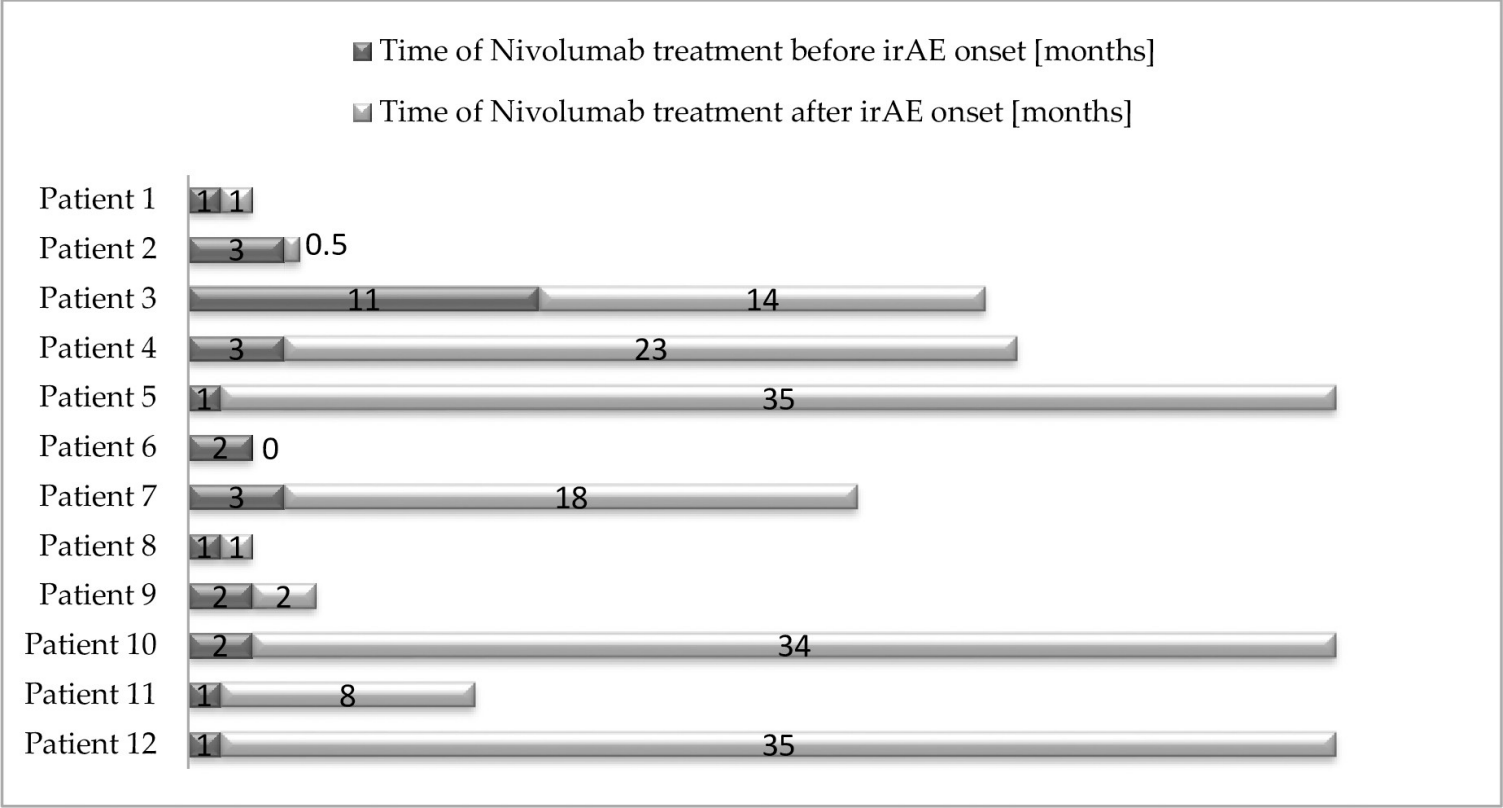
Landmark analysis of patients with endocrine irAE. (Patient number 6 developed irAE 2 weeks after treatment termination).

The characteristics of patients who developed endocrine disorders most probably in the immunological mechanism (irAE group, n = 12, 34.3%) are presented in Table 2 and Fig 1. The median onset time of any endocrine irAEs was 2 months (range 1–11 months). The median time onset of thyroid dysfunction was 2 months (range 1–3 months).

**Table 2:** Endocrine irAEs among examined patients.

No	Sex/ age	Type of NSCLC^1^	irAEs	CTCAE Grade [7]	Onset time (months)	Treatment	Outcome	Anti thyroid antibodies^2^
1	F/64	Adeno	Hyperthyroidism^3^	2	1	Observation→ Levothyroxin	Hypothyroidism	↑
2	F/58	Squamous	Hypothyroidism	1	3	Observation	Resolution	↑
3	F/79	NOS	Hypophysitis	3	11	Steroids → hormonal replacement therapy (Hydrocortisone Levothyroxine)	Secondary hypothyroidism, Secondary insufficiency adrenal glands, Secondary hypogonadism	N
4	M/75	Squamous	Hyperthyroidism	2	3	Thiamazole	Hyperthyroidism	N
5	F/65	NOS	Hyperthyroidism^3^	2	1	Observation→ Levothyroxin	Hypothyroidism	↑
6	M/65	Squamous	Hyperthyroidism	2	2	Steroids^4^	-	ND
7	M/81	Squamous	Hypothyroidism	2	3	Levothyroxin	Hypothyroidism	↑
8	F/67	Adeno	Hyperthyroidism^3^	1	1	Observation	Resolution	ND
9	F/63	Squamous	Hypothyroidism	2	2	Levothyroxin	Hypothyroidism	ND
10	M/54	Squamous	Hyperthyroidism^3^	2	2	Observation→ Levothyroxin	Hypothyroidism	N
11	M/66	NOS	Hyperthyroidism^3^	2	1	Observation→ Levothyroxin	Hypothyroidism	↑
12	M/52	Squamous	Hyperthyroidism^3^	2	1	Thiamazole→ Levothyroxin	Hypothyroidism	↑

^1^ squamous, squamous cell carcinoma; adeno, adenocarcinoma; NOS, not otherwise specified^;^

^2^ ↑, elevated; N. negative; ND, no data

^3^ clinical presentation and the natural history of this disorder suggest destructive thyroiditis

^4^ The key indication for systemic glucocorticosteroids in this case was immunological pneumonitis.

In the examined group, hyperthyroidism was observed in eight patients (22.9%); in six of them, thyroid dysfunction was transient, most probably due to destructive thyroiditis. In three patients, hypothyroidism was observed manifesting either the aggravation of pre-existing Hashimoto’s disease or reflecting de novo development of autoimmune thyroiditis (yet, thyroid antibodies were stated in only some of the patients).

In the group of patients with thyroid disorders, persistent hypothyroidism eventually developed in seven cases (58.3%) and normal thyroid function was restored in two cases (16.6%); in one case, subclinical hyperthyroidism remained. One patient died because of pulmonary adverse effects.

In one patient (female, 79 years), 11 months after nivolumab therapy initiation, patient reported difficulties in reading, weakness, worse well-being, and weight loss. In laboratory studies hyperkalaemia and hyponatraemia was reported. In the hormonal assessment, secondary hypothyroidism was detected and diagnostics of pituitary function were performed. Eventually, panhypopituitarism and hyperprolactinemia (due to compression of stalk) and temporal visual field defects were diagnosed. The MRI revealed a 26 × 16 × 14 mm pathological mass and irregular contrast enhancement, an enlargement of sella turcica with suprasellar expansion and stalk thickening. The diagnosis of autoimmunological hypophysitis was established (Grade 3); in effect, immunotherapy was interrupted. Replacement therapy with hydrocortisone and levothyroxine was initiated. When the irAEs diminished (Grade 1), the immune therapy was resumed. The regression of the pituitary lesion regression was observed (16 × 10 × 15 mm) in the control MRI performed six months later [31]. Secondary hypothyroidism, mild hyperprolactinemia, and secondary insufficiency of adrenal glands were maintained to the end of observation of the patient (as well as insufficiency in gonadal and growth hormone axis, but these were not treated).

The analyzed endocrine irAEs ranges in severity from mild to moderate (Grade 1–2). Only the case of hypophysitis was estimated as Grade 3. The information on the introduced treatment is presented in Table 2.

When comparing the two groups of patients (Table 1), no relation was found between endocrine irAE development and patient sex, age, treatment protocol, stage of the disease, smoking status, age at the disease onset, treatment time, presence of *EGFR* mutation, *ALK* rearrangement, or the reason for treatment discontinuation. A higher incidence of adenocarcinoma in the non-irAE group was found (p = 0.011). Previous radiotherapy (history of palliative tumor radiation before immunotherapy introduction) was more common (close to significant) in patients with irAEs (p = 0.07).

No differences were identified in terms of PD-L1 expression between the groups, yet these data were available for less than half of the patients.

### Survival probability analysis

**Fig 2:**
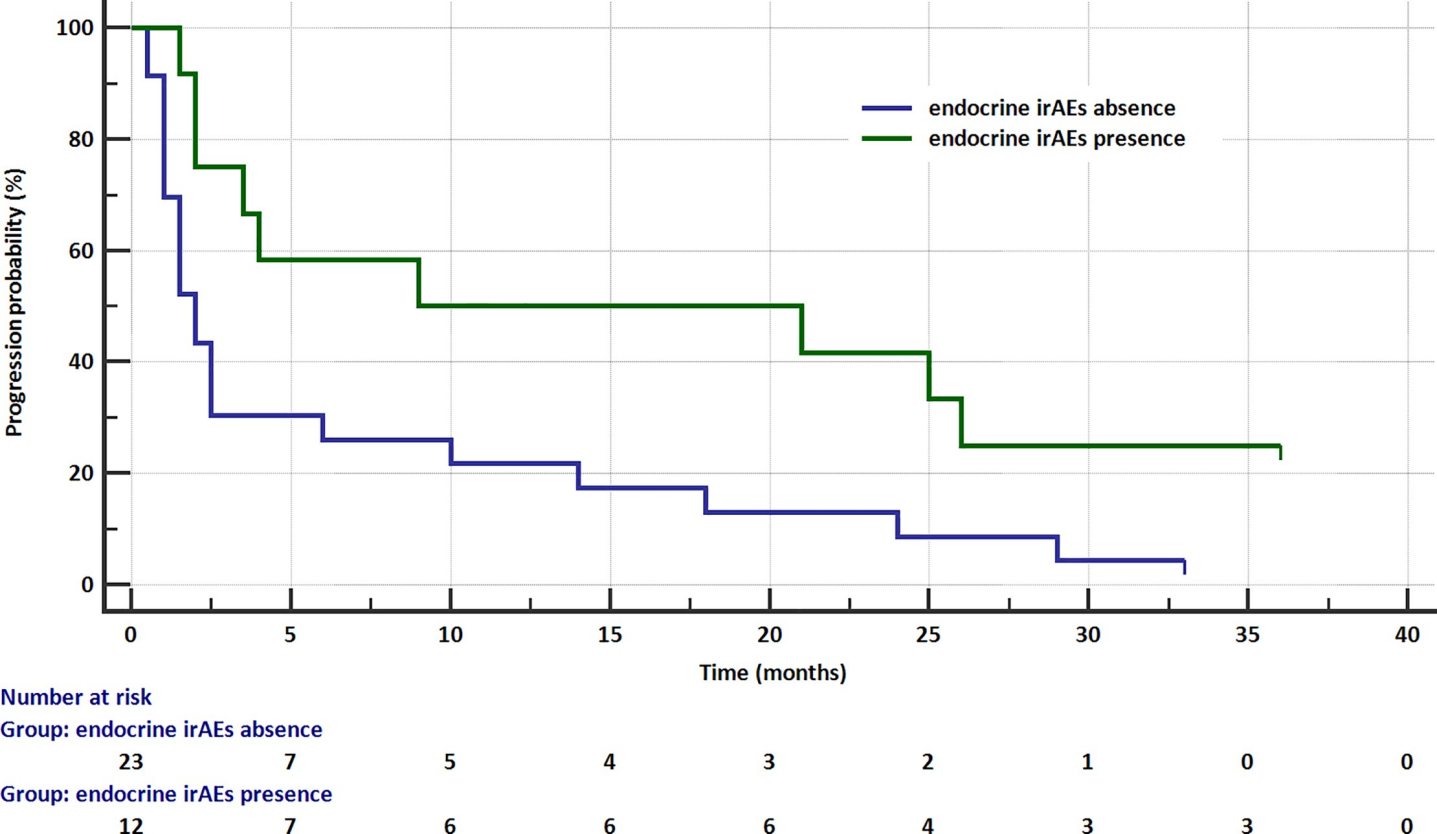
Kaplan–Meier PFS curve in patients with and without endocrine irAEs.

To evaluate the potential influence of endocrine irAEs on patients’ prognosis, the two groups were compared in terms of progression free survival (PFS). Median progression free survival, according to Kaplan-Meier survival analysis, in the patients without endocrine adverse events was 2 months (95%CI: 1.50–2.50), while for patients with endocrine events was 9 months (95% CI: 3.50–26.0). The occurrence of endocrine complications statistically significantly reduced the risk of disease progression (HR = 0.415; 95% CI: 0.196–0.878; p = 0.0214; Fig 2 corrected). Patients with endocrine irAE were treated significantly longer with nivolumab and they received more doses of nivolumab (p = 0.009) in comparison to the patients who did not develop endocrine irAEs.

**Fig 3:**
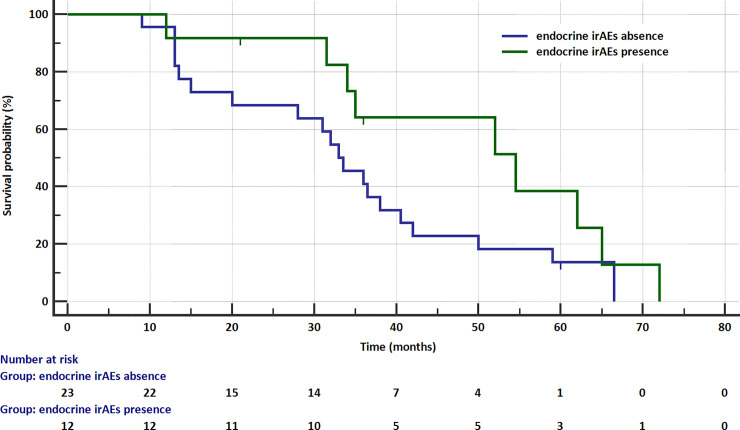
Kaplan-Meier overall survival curve in patients with and without endocrine irAEs.

In the term of overall survival (OS), patients with endocrine events had shown insignificantly longer OS median (54.5 months), when compared with those without endocrine adverse events (33.5 months). Moreover, the presence of endocrine events had insignificantly impact on reduced risk of survival (HR = 0.522; 95% CI: 0.243–1.120; Fig 3).

Of 35 examined patients, 6 continued nivolumab therapy for at least two years, progressing into partial remission with good tolerability of the therapy. At the time of data collection, three of them were continuing nivolumab therapy (36 months from treatment initiation) and were in good general condition. No patient died or discontinued nivolumab treatment because of hormonal disorders: in most of the cases, observation or symptomatic treatment was sufficient.

To minimize the possibility of the influence of the time that has passed since the start of the study and the classification the subjects into the group experiencing complications (immortal time bias) Cox analysis was performed. Affiliation to the group of people with irAE was the time-dependent covariance variable. Table 3 presents the results of the conducted analysis.

**Table 3:** Time to progression and experience of endocrine complications on nivolumab treatment.

	B	SE	Wald	Df	p	Exp(B)	95% CI dla Exp(B)	
							LCI	UCI
Time-dependent covariance variable	-0,264	0,467	0,318	1	0,573	0,768	0,308	1,920
Model summary	-2LLg = 179,922; χ ^2^ = 0,325; df = 1; p = 0,569							

The conducted analysis did not show that the proposed model explain the variability of the time to progression. It has not been shown that subjects with endocrine complications experienced progression later than subjects without them.

## Discussion

Our study was a single-center, retrospective study on the incidence and nature of endocrine adverse effects during immunotherapy for advanced NSCLC. The incidence of endocrine irAEs was high (34.3%), with thyroid disorders being the most common. The prevalence of endocrine disorders was higher than in clinical trials (up to 14.2%) [5, 32–34] but consistent with more recently published studies (up to 39%) [13, 35–38]. In other studies covering lung cancer, this percentage was estimated as 19.5% [12, 19, 22, 32, 33, 39, 40]. In the analysis performed by Judd et al., this incidence was 23%. However, among these patients, 77% were treated with nivolumab; the remaining patients received a PD-1 inhibitor, pembrolizumab [41]. The differences might be the result of different diagnostic criteria for particular hormonal disorders, and early, repeated evaluation of thyroid dysfunction during treatment. The risk of immunological disorders during ICIs treatment is elevated. Treatment with PD-1 and PD-L1 inhibitors is particularly related to thyroid function disorders. In the meta-analysis performed by Abdel-Rahman et al., the relative risk associated with immune checkpoint inhibitors versus controls was 8.26 (4.67–14.62) for all-grade hypothyroidism and 5.48 (1.33–22.53) for hyperthyroidism, with no significant differences related to the type and dose of treatment [42].

The most common pathomechanism of thyroid function disorders during PD-1 inhibitors treatment is destructive thyroiditis with transient hyperthyroidism or thyrotoxicosis, followed by thyroid function normalization or hypothyroidism development [12, 14, 32, 33, 43, 44]. This agrees with the results obtained in our study. In our observation, similar to other studies [40], no case of Graves’ disease was reported.

Several studies have proved that underlying thyroid dysfunction is a risk factor of endocrinological adverse events [13, 27, 45–47]. Our study also included patients with a medical history of endocrine disease prior to treatment; in the case of indifferent nodular goiter, there is usually no exacerbation; in the case of autoimmune thyroid disorders it is often the case.

The basis of the mechanism in thyroid disorders is the increased cellular response according to the action of the PD-1 molecule on the activated immunocompetent cells. This is often accompanied by a humoral component, expressed by the presence of anti-thyroid antibodies. In the present study, antibodies were elevated in only part of the irAE group; in some of them, the assay was not available, which complicated inference. The presence of antibodies in literature ranges between 16% and 80% [14, 27, 44, 48], however in some studies the presence of the antibodies was uncommon [49–52]. The presence of anti-thyroid antibodies prior to immunotherapy is a risk factor for the development of a gland dysfunction [4, 27, 46]. The antibodies appear soon after anti-PD-1 drug administration, suggesting that the therapy probably unveils a thyroid inflammation that existed prior to the therapy but in a latent form [27]. These observations suggest a complex mechanism of irAEs in the thyroid, including an antibody-independent mechanism of the cellular response or the role of other thyroid antibodies that are not evaluated routinely. It remains to be determined whether the appearance of thyroid antibodies in the course of the treatment causes thyroid inflammation or if it is rather the result of the humoral response to the presence of thyroid antigens in the course of gland destruction [49, 53]. The dynamics of thyroiditis following treatment with anti PD-1 inhibitors differs from the typical Hashimoto’s thyroiditis, which usually follows a gradual course. Immunotherapy-induced inflammation usually begins within the first months of treatment. The thyrotoxicosis phase is often followed by hypothyroidism. In Hashimoto’s disease, the severity of hypothyroidism is gradual, and the thyrotoxicosis phase is rarely observed. This confirms the different and not entirely clear mechanism of thyroid dysfunction induced by ICI treatment [27, 49].

An important aspect in the field of irAE is the stratification of patients in terms of the risk of their occurrence. At present, no clear recommendations regarding monitoring of the thyroid function are available, apart from the recommendation of periodic (usually every second drug administration) control of thyroid hormones. Routine assessment of thyroid antibodies before starting immunotherapy could help identify patients at increased risk and would facilitate further monitoring [43].

Hypophysitis is a rare complication in nivolumab monotherapy (up to 0.6%) [5, 20]. In our study, this adverse effect was diagnosed in one patient, hypopituitarism was found in the thyroid, cortico-, growth hormone and gonadotropic lines with mild hyperprolactinemia with the onset of symptoms after 11 months of treatment [31]. Other studies also reported cases of patients with isolated ACTH deficits during nivolumab therapy, where symptoms developed after 12–13 doses administrations [54–57]. The meta-analysis of Abdel-Rahman et al. disputed the greater risk of this condition during treatment with nivolumab [42]. More commonly, the complication is observed in the course of anti-CTLA-4 therapy [39]. This can be explained by anti-CTLA-4 therapy stimulating autoreactive T cells as well as the production of antibodies directed against pituitary antigens, and enhanced complement activation. As CTLA-4 may be expressed on pituitary cells, it becomes a target for anti-CTLA-4 antibodies [58]. The expressions of both RNA and CTLA-4 protein have been found, particularly in lacto- and thyrotropic cells of the pituitary gland [58]. The mechanism of hypophysitis in the course of nivolumab therapy remains unknown. A typical feature observed with MRI is the moderate enlargement of the pituitary, enlargement of the stalk, and homogeneous contrast enhancement [21, 59]. In our patient, non-heterogenous contrast enhancement was present, but MRI and clinical features and dynamics in follow-up MRI examination definitely pointed to inflammation. Non-homogenous contrast enhancement was described in the literature as an atypical feature [59], but Kurokawa found pituitary geographic hypoenhancing lesions in most analyzed cases of hypophysitis due to ICI treatment [53]. Enlargement of the sella turcica needs to be differentiated with rare pituitary metastasis from melanoma, breast, and lung cancer and macroadenomas [59, 60], but in the last mentioned case, asymmetry or focally enlarged pituitary with normal stalk is significant [59]. The recovery of the pituitary–thyroid and pituitary–gonadal axis can occur. However, improvement in the pituitary–adrenal axis has been observed in very few cases [52].

Similar to other studies [29, 34, 44, 48, 61, 62], our patients were mostly asymptomatic or oligosymptomatic (Grade 1–2), and the number of Grade ≥ 3 AE’s was low. Only one patient with pituitary gland inflammation required systemic steroid therapy. Management of irAE’s includes the use of prednisone or the equivalent in anti-inflammatory doses (0.5–2 mg/kg) from Grade 2 for pituitary inflammation and in Grade 3–4 for adrenal insufficiency [5, 9, 63–65]. Recommendations regarding thyroid dysfunction include levothyroxine treatment for persistent hypothyroidism and symptomatic treatment for hyperthyroidism/thyrotoxicosis, usually without the need for immunosuppression and without the need to modify the dose of nivolumab [5, 9, 63–65]. Destructive thyroiditis with a phase of transient hyperthyroidism, which is estimated in the literature at 3.2–6.5% [15], often quickly passes in the hypothyroid phase, observed up to 16 to 32 days after the last documented TSH suppression [66]. Thyreostatic drugs are rarely applicable in this situation. In our group, only one patient required anti-thyroid medication. The data in the literature suggest that the use of steroids in hyperthyroidism/thyrotoxicosis could prevent persistent hypothyroidism. This requires further research [43, 62].

In our study, the median occurrence of endocrine disorders was 2 months, which agrees with the literature data [36, 40, 46]. In other studies, the median time of onset was estimated as ranging from 5.3 to 12 weeks [44, 66]: hypothyroidism at 2.9 months and hyperthyroidism at 1.5 months [5]. The occurrence of thyroid dysfunction among the analyzed patients was found as early as one month after treatment. This early onset of irAEs suggests the existence of autoimmune thyroid disease in the latent phase before the inclusion of immunotherapy, indicating that immunotherapy only accelerated clinical the manifestation of dysfunction.

In the studied population, no relationship was found between the risk of irAEs and demographic factors, which corresponds to other types of studies [4, 12, 46], although some reports indicated different functions of the thyroid in women [43]. In the retrospective study by Campredon et al. involving 105 patients with NSCLC, the patients in the thyroid dysfunction group were significantly younger with a female predominance [40]. In the literature, there is no correlation between irAEs performance and the histological type of cancer, history of smoking, or line of treatment with nivolumab [46]. Similar results were obtained in this study. The incidence of irAEs was lower among adenocarcinoma patients, which requires further research.

In the irAEs group, we observed a trend toward more frequent radiotherapy (RT) prior to nivolumab treatment. We found no correlation between radiotherapy and the occurrence or severity of irAE risk, however, reports state that irradiation can modulate the immune response, e.g., by increasing the expression of MHC I antigens and other adhesion molecules on tumor cells and thereby increase their immunogenicity [67–69]. The effect also accompanies the regression of metastatic changes outside the area of radiation activity [67]. Radiation therapy can also affect the up-regulation of PD-1 and CTLA-4 molecules on tumor cells [67, 70]. Preclinical studies suggested that complete RT increases the effectiveness of ICI [70]. There are reports indicating an increase in risk of pneumonitis in patients who had previously undergone radiation therapy in this area [71], which was also a concern in our patients [67, 72, 73]. Several analyses have considered the possibility of combining these methods of treatment or even more extensive use of immunotherapy in patients who need radiotherapy [67–69]. In the KEYNOTE-001 study with pembrolizumab, Shaverdian et al. found longer PFS in the group previously treated with radiation [72]. These results suggest synergistic effect of prior radiotherapy and ICI treatment, although further research is required in this area.

Significantly higher PFS in the irAE group was found in the analyzed population. The literature also reports a positive effect on survival rates or response to cancer treatment in patients who experienced irAEs [11, 27, 38, 41, 46, 61, 74–78]. In the presented study, based on the Cox proportional hazard model relationship between longer progression free survival and the presence of irAE was not found, although it might be the result of the small study group. Moreover, irAE are characterized by the fact that they may occur late, even after the end of immunotherapy, which may also influence the result of this type of analysis. In Yamazaki et al., patients with NSCLC who developed thyroid dysfunction received significantly more doses [12], which is consistent with the results of this study. This issue is related to the assessment of patient prognosis. The presence of anti-thyroid antibodies and the development of irAEs have been recognized as independent prognostic factors for favorable response to treatment [46]. In Ricciuti et al., patients who developed at least two irAEs during treatment had a significantly longer median PFS and overall survival (OS) compared to those with one or no AEs [37]. Conversely, in Freeman-Keller et al., only the presence of cutaneous irAEs was a positive prognostic factor. The study population included a small group of melanoma patients, which might partly explain the finding [75]. A favorable effect on prognosis in patients who developed irAEs was particularly noticeable in cases of early onset irAEs [11] and within the scope of asymptomatic or low-severity complications [41]. However, Yamauchi described a stronger relationship between better prognosis in cases of overt thyroid disorders than subclinical ones [38]. In turn, Ksienski et al. did not confirm the association of any irAE, including cutaneous, endocrine, pulmonary, or hepatic irAEs, with the improvement in survival rates, whereas gastrological complications (colitis) were associated with worse prognosis [79]. Yamauchi et al. described significant higher OS probability at 12 months’ treatment in a thyroid irAE group among lung cancer patients [38]. The postulated association of irAEs with the effectiveness of NSCLC treatment is the greater intensity of the immune response, including activity against cancerous tissue. Some authors suggested a common target antigen in lungs and in the thyroid [38], however, this requires further research.

Higher PD-L1 expression in tumor tissue is associated with a better response to treatment [32, 80, 81], although some studies do not prove this association [33]. In this study, this relationship was not confirmed. Some studies determined the relationship between PD-L1 expression and the risk of irAEs [11, 28, 80, 81]. It appears that the status of PD-L1 expression on tumor cells is not a prognostic factor for irAEs, and it was also not relevant in this study. Our study has several limitations: its retrospective nature, the randomization, and being a single-center study. However, our aim was to assess patients in real-world conditions. PD-L1 expression was not determined on all patients, nor were thyroid antibodies assessed on all patients. Another limitation results from the small study group, which decreases the ability to draw causal relationships. Among patients enrolled in this analysis were those with EGFR mutation, who often respond poorly to checkpoint inhibitors. However, these patients were part of the population that received nivolumab in real -life conditions (due to an early access treatment program). Excluding patients with EGFR mutation would have decreased the size of the study subpopulations, preventing comparison. In the future, we will continue to expand the study group to include treatment with other PD-1 inhibitors. We postulate that this study provides a valuable assessment that may impact everyday practice. The trend of a higher frequency of irAEs among patients who have undergone radiation therapy needs to be explored and confirmed in further studies.

## Conclusions

Nivolumab therapy is associated with an increased risk of endocrine adverse effects, particularly thyroid dysfunction, which implies the need for hormonal control during the therapeutic process. Endocrine adverse effects can be successfully treated pharmacologically and usually do not require discontinuation of immunotherapy. The relationship between a better cancer prognosis in patients who developed endocrine irAE has not been found.
